# Changes in endogenous gene transcript and protein levels in maize plants expressing the soybean ferritin transgene

**DOI:** 10.3389/fpls.2013.00196

**Published:** 2013-06-14

**Authors:** Milly N. Kanobe, Steven R. Rodermel, Theodore Bailey, M. Paul Scott

**Affiliations:** ^1^Department of Agronomy, Iowa State UniversityAmes, IA, USA; ^2^Department of Genetics, Development and Cell Biology, Iowa State UniversityAmes, IA, USA; ^3^Department of Statistics, Iowa State UniversityAmes, IA, USA; ^4^USDA-ARS, Corn Insects and Crop Genetics Research UnitAmes, IA, USA

**Keywords:** soybean ferritin, transgenic maize, endogenous genes, gene expression, transcription, iron

## Abstract

Transgenic agricultural crops with increased nutritive value present prospects for contributing to public health. However, their acceptance is poor in many countries due to the perception that genetic modification may cause unintended effects on expression of native genes in the host plant. Here, we tested effects of soybean ferritin transgene (SoyFer1, M64337) on transcript and protein levels of endogenous genes in maize. Results showed that the transgene was successfully introduced and expressed in the maize seed endosperm. mRNA abundance of seven tested iron homeostasis genes and seed storage protein genes differed significantly between seed samples positive and negative for the transgene. The PCR negative samples had higher zein and total protein content compared to the positive samples. However, PCR positive samples had significantly higher concentrations of calcium, magnesium, and iron. We have shown that the soybean ferritin transgene affected the expression of native iron homeostasis genes in the maize plant. These results underscore the importance of taking a holistic approach to the evaluation of transgenic events in target plants, comparing the transgenic plant to the untransformed controls.

## Introduction

Genetic engineering has been used to improve the performance and nutritional qualities of many economically important crops world-wide. Maize (*Zea mays*) is no exception and since it is a major source of dietary calorie intake in many countries, a lot of research focusing on its nutritional enhancement has been done. With most of the world's population dependent on cereal crops as basal sources of their diet (Minihane and Rimbach, 2002), it is imperative that these crops contain adequate levels of nutrients required for good health.

Iron is one of the major micronutrients required in the human body. However, cereals are very poor sources of iron as it is not readily bioavailable. This is to a large extent due to the presence of phytates that bind with dietary iron, making it unavailable for absorption (Hallberg et al., 1989; Minihane and Rimbach, 2002). Cereal grains contain 0.2–2.8 mg/100 g of iron (Glahn et al., 2002) but less than 2% is absorbed in the human gut. Therefore, increasing the quantity and bioavailability of iron in cereals presents one way to fight anemia-related problems, especially in populations where cereals are a major staple crop. This could be achieved through classical breeding or by genetic engineering. However, traditional breeding is limited by inadequate genetic variation for iron content and bioavailability in the natural maize populations.

Genetic manipulation through over expression of ferritin genes from different sources as a means to increase the amount of iron in plants has been successful (Goto et al., 1999; Drakakaki et al., 2000, 2005; Lucca et al., 2001, 2002; Vasconcelos et al., 2003; Qu et al., 2005; Aluru et al., 2011; Borg et al., 2012). While these results are promising from the nutritional perspective, only one of these studies examined gene expression and transcription profiles of native genes in ferritin-transformed plants compared to non-transformed plants. As such, the impact of foreign genes on expression and transcription of native genes remains relatively uncharacterized in this system. In Arabidopsis, El Ouakfaoui and Miki (2005) reported a small number of genes differing in expression levels between T-DNA-insertion mutants and their non-transformed counterparts. On the other hand, Baudo et al. (2006) compared changes in mRNA levels of conventionally generated and genetically modified wheat lines expressing glutenin with the traditional wheat lines using 9K cDNA microarray technique and they concluded that breeding through conventional means causes more variation in expression levels than using genetic engineering.

Introduction of genes involved in iron storage is likely to alter the regulation of expression and transcription of native genes for iron storage or those genes required for other iron homeostasis processes. In transgenic tobacco expressing soybean ferretin, for example (Van Wuytswinkel et al., 1999; Vansuyt et al., 2003) physiological and biochemical changes were observed that mirrored iron-deficient plants, possibly due to sequestration of iron in ferritin. Knowledge of the transgene effect on expression of native genes in the host genome is required in order to predict unintended effects of these foreign genes on the levels and stability of the native genes. If event evaluation and selection is focused on identifying presence of superior transgene phenotypes, it is possible that internal changes in expression levels of other genes within the host genome as a result of transformation will be overlooked.

In maize, the 27 kDa zein promoter has been used to regulate endosperm specific expression in transgenic cereal crops and indeed, the same promoter was used in this study to drive the expression of the soybean ferritin gene to the maize seed endosperm. Since maize contains endogenous zein storage proteins, it would be interesting to examine the effect of the introduced transgene driven by the zein promoter on expression and transcription of other endogenous genes involved in iron sequestering and storage as well as endogenous zein protein genes in the maize seed endosperm, since the zeins have been reported to share promoter regions (Kodrzycki et al., 1989). Our main objective was therefore to over express the soybean ferritin gene in maize seed endosperm and study the transgene effects on the transcript and expression levels of selected endogenous maize genes in roots, leaves and seed endosperm.

## Materials and methods

### Construct development and transformation

**Figure 1:**
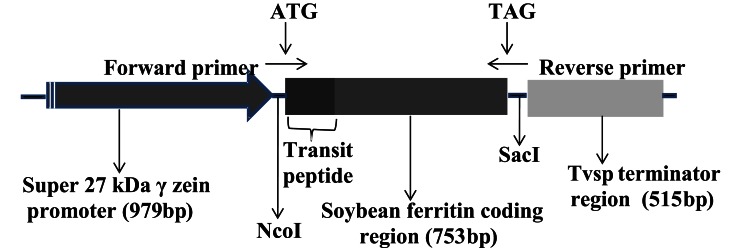
**Organization of the soybean ferritin transformation construct.** The construct consisted of the super gamma zein promoter, the soybean ferritin coding sequence and the Tvsp terminator from soybean vegetative storage protein gene. The corresponding sizes for each construct component are indicated in brackets below the construct component name. The ATG and TAG indicate the start and stop sites, respectively, while NcoI and SacI represent the enzyme restriction sites flanking the soybean ferritin sequence with the plastid transit peptide. The forward and the reverse primer directions are indicated by arrows pointing in opposite directions.

The soybean ferritin construct (pMNK01) is the same construct used in Aluru et al. (2011) and consisted of the super gamma zein promoter sequence (979 bp) (Aluru et al., 2008) for endosperm-specific expression, the soybean ferritin coding sequence (753 bp) with a plastid transit peptide to direct protein accumulation to the amyloplast (Lescure et al., 1991), and the Tvsp terminator sequence (515 bp) from the soybean vegetative storage protein gene (Aluru et al., 2008). The construct was developed using the polymerase chain reaction (PCR)-based cloning and it is illustrated in Figure 1. Plasmid delivery into maize HiII line (A188 × B73) (Armstrong et al., 1991) was done by the Iowa State University Plant Transformation Facility according to Frame et al. (2000). The plasmid construct pMKN01 was co-bombarded with pBAR184 (Frame et al., 2000), which has the maize ubiquitin promoter that drives the expression of the *Streptomyces hygroscopicus* gene for the bialophos resistance to enable selection of successful transformation events. Successful transformations were confirmed in biolophos-resistant calli by PCR, using primer pairs specific for the soybean ferritin gene [5′GCCATGGCTCTTGCTCCATCC3′ (forward primer) and 5′CAAAGTGCCAAACACCGTGACCC3′ (reverse primer)]. The plantlets regenerated were transferred to the greenhouse, grown to maturity and the seeds were harvested and separated by ear. A total of eight transformation events were harvested after bombardment and selection at the callus level.

### Greenhouse and field production of transgenic inbred lines

Plants regenerated from the transformed callus were crossed to the *Zea mays* L. inbred B73 to produce F_1_ seeds. The F_1_ plants were backcrossed to B73 to produce BC_1_F_1_ seeds for the second generation. The BC_2_F_1_ and BC_3_F_1_ generation seeds were produced by backcrossing the BC_1_F_1_ and BC_2_F_1_ generation plants to B73 in subsequent third and fourth generations, respectively. Thus, the genomic composition of all the seeds planted for analysis was 93.75% B73 by pedigree, with the remainder being from A188. The selection of which plants to advance to the next generation was done based on presence and expression of the soybean ferritin transgene in individual seeds. The first two generations were grown in a greenhouse, while the third and fourth generations were grown in the field at the Iowa State University's transgenic nursery at Woodruff farm near Ames, Iowa.

### Field plot design for production of maize plant tissues

Tissues used in zein and mineral analyses were produced in the field in 2009 while those for DNA detection, western blots and mRNA transcript analyses were produced in the field in 2010. Each transformation event was randomly assigned to a plot. A plot consisted of four rows. The first three rows contained PCR positive seeds planted ear to row from three different ears within the same transformation event. The fourth row contained a bulk of PCR negative seeds from the same three ears.

### Ferritin DNA detection in transformed maize seed endosperms

Four transformation events (P344-2-4-1, P344-4-1-6, P344-5-2-1 and P344-6-6-1) were selected and used in this analysis. PCR was used to test for presence of soybean ferritin DNA in individual seeds. Test samples were obtained using a non-destructive method where a 10 mg sample of the endosperm was drilled from each of the seeds. DNA was extracted from each sample using the phenol-chloroform manual extraction method. The extraction buffer consisted of 200 mM Tris-HCl (PH 7.5), 250 mM NaCl, 25 mM EDTA, and 0.5% SDS, all in water. The PCR reaction was performed with the EconoTaq plus Green 2x master mix (Lucigen Corporation, Middleton, WI) following the manufacturer's instructions. The primers used were designed to amplify a 753 bp soybean ferritin fragment and these were 5′GCCATGGCTCTTGCTCCATCC3′ (forward primer) and 5′CAAAGTGCCAAACACCGTGACCC3′ (reverse primer). The PCR reaction cycle consisted of initial denaturation for 2 min at 95°C, denaturation for 30 s at 95°C, annealing for 30 s at 57°C and extension for 1 min at 72°C for a total of 35 cycles. The PCR products were run on an 1% agarose gel containing ethidium bromide which was photographed under UV light.

### Comparison of soybean ferritin protein in transgenic maize seeds positive or negative for the soybean ferritin DNA

Western blot analysis was carried out to determine the expression of the soybean ferritin transgene in maize seed endosperm. Mature transgenic seeds were dried and a 10 mg sample was drilled from individual seeds and collected into a 1.5 ml tube. The remaining portion of each of the seeds was kept separate. DNA was extracted from the samples and PCR performed as described in the previous section. The PCR positive and negative samples were noted and their seeds separated. An endosperm sample was drilled from the saved portion of the seed and used for protein extraction and detection. Total protein was extracted with 100 μl of extraction buffer (200 mM Tris-HCl pH 8.0, 100 mM NaCl, 400 mM sucrose, 10 mM EDTA, 14 mM 2-mercaptaethanol and 0.05% Tween-20) for a 10 mg sample of the endosperm powder. 20 μl of total protein were analyzed by electrophoresis using sodium dodecyl sulphate (Laemmli, 1970) on a 12% polyacrylamide gel. After the run was completed, the proteins were transferred to a nitrocellulose membrane for 2 h using a biorad transblot apparatus according to the manufacturer's directions. Polyclonal antibodies for the soybean ferritin gene were raised in rabbit against soybean ferritin peptide (PQVSLARQNYADEC) produced by GenScript Company, Piscataway, NJ. Protein detection was performed using standard western blot analysis protocols (Anti-rabbit-AP secondary antibody with AP conjugate substrate, Biorad Laboratories, Hercules, CA) following the manufacturer's instructions. The blot was probed with 1:300 and 1:3000 dilutions of the primary and secondary antibodies, respectively.

### Comparison of transcript levels of maize endogenous genes in maize plant tissues positive or negative for the soybean ferritin transgene

The aim of this experiment was to compare mRNA transcripts of endogenous maize genes in roots, leaves and seed endosperms using a quantitative polymerase chain reaction (qPCR) method. The presence or absence of the soybean ferritin transgene in maize plants was first determined by PCR using primers specific for the transgene (see previous sections). Transcript levels of PCR positive plants were compared to those of PCR negative plants in maize roots, leaves and seed endosperm tissues. Maize plants in BC_2_F_1_ and BC_3_F_1_ generations segregating for the soybean ferritin transgene were grown in the field in 2009 and 2010, respectively. The first leaf and root samples were collected from individual plants 1 month after planting (MAP) and at 18 days after pollination (DAP), leaf, root, and seed endosperm samples were collected. The 1 MAP and 18 DAP data collection points were included in the analysis in order to capture differences in mRNA transcript levels of endogenous maize genes before and after onset of soybean ferritin transgene expression, respectively. Leaf and root samples were collected from one plant in each PCR positive row and three plants from each PCR negative row for a total of six plants per transformation event for each of the four events (P344-2-4-1, P344-4-1-6, P344-5-2-1, and P344-6-6-1). Similarly, twenty immature seed endosperms were collected from each of the three ferritin PCR positive and negative ears from six plants for each of the four transformation events. Each endosperm sample was divided into two portions, one for DNA analysis in order to identify positive and negative endosperms and the second for mRNA analysis. Tissues were immediately frozen in liquid nitrogen while in the field and upon arrival in the laboratory, they were stored at −80°C until processing. DNA was extracted from each of the endosperms and subjected to PCR analysis for soybean ferritin transgene detection. Soybean ferritin positive and negative seeds were identified and mRNA was extracted from the saved endosperms using the PolyATract mRNA Isolation System kit (Promega Madison, WI). Likewise, mRNA was extracted from the root and leaf tissues using the same method as above. Quantitative PCR analysis was performed with the Brilliant II SYBR Green QRT-PCR Master Mix kit (Stratagene, Santa Clara, CA).

#### Genes used for the analysis

A complete list of genes and tissues in which these genes were tested in addition to the primer information for each gene is presented in Table 1. Among the selected genes were the zeins, the major group of seed storage proteins genes in the maize seed endosperm. These included 27 kDa γ and 16 kDa γ-zeins, 15 kDa β-zein, 19 kDa and 22 kDa α-zeins, and 18 kDa δ zein. Also, genes involved in the iron homeostasis pathway (Maize ferritin, maize ferredoxin-1, *Zea mays* 4 iron—4 sulphur ferredoxin (ferredoxin), *maize nicotianamine amino transferase* 1 (*ZmNAAT1*), *maize yellow stripe* 1 (*ZmYS1*), *maize nicotianamine synthase* 1 (*ZmNAS1*), *maize nicotianamine synthase* 2 (*ZmNAS2*) and *maize nicotianamine synthase* 3 (*ZmNAS3*) were included in this study. These genes were chosen for this study because they are known to respond to or be involved with determining iron homeostasis and are well-characterized in maize. The reference gene used for normalization was the 18S ribosomal RNA.

**Table 1:** **Details on gene, primer information, and maize plant tissue in which the gene was tested in the QPCR experiment**.

**Gene name**	**Accession no.**	**Maize plant tissue tested**	**Sequence (5′–3′)**	**Product (bp)**	**References**
27kDa γ-zein	EU976420	Seed endosperm	CCACCATGCCACTACCCTAC ACTGATGCCTCAGGAACTCG	166	Alexandrov et al., 2009
16kDa γ-zein	AF371262	Seed endosperm	TGGAGAACTCGACACCATGA GGTGGTTGAGTGGGGTATTG	201	Woo et al., 2001
15kDa β-zein	AF371264	Seed endosperm	TCAGTAGTAGGGCGGAATGG TGTACGAGCCAGCTCTGATG	179	Woo et al., 2001
19kDa α-zein	AF371269	Seed endosperm	CGTGGGTGAGACCAACTAGC GAAGACACCGCTGGTGAGAG	203	Woo et al., 2001
18kDa δ-zein	AF371265	Seed endosperm	CTCTGATTCCATCTCGCACA GGCATGCCGACTTCATTATT	161	Woo et al., 2001
22kDa α-zein/Floury2	L34340	Seed endosperm	CAACAGTTTCTGCCATCACTG GGCTAGTTGACTGAGCACTGG	162	Coleman et al., 1995
Soybean ferritin	M64337	Seed endosperm	AACTGCTCCCCAAGTCTC G CAGCGTGCTCTCTTTCTTCC	200	Lescure et al., 1991
Maize ferritin	X61392	Root, leaf, Seed endosperm	CGACCCCCACGCGCCTATATC GCGGCCCCACCAGAGAGATG	200	Lobreaux et al., 1992
ferredoxin-1	EU958223	Root, leaf, seed endosperm	GCCTGTCGTCGCAGTAGTGA AGGAGTAGGGCAGGTCCAAC	172	Alexandrov et al., 2009
4Fe-4S ferredoxin	EU969603	Root, leaf, seed endosperm	AGAGCTGTGTCCTATGTTCGAG TAACTGCAACCAGCTTCACG	145	Alexandrov et al., 2009
Zm*NAAT1*	AB375372	Root	TGGCCACATTGCTCCTGTCTTG GAAGTGCTCCCTGAAAGTTGCTGA	184	Inoue et al., 2008
yellow stripe 1	NM_001111482	Root	GATGCAAGTCCGAGGGTTCCTC CGAGGTGCGAACAGATCATCCC	180	Ueno et al., 2009
ZmNAS1	AB061270	Root, leaf, seed endosperm	TGTTCACCAGCCTCGTCATG CTGAAGTAGGGAAAGCGGCC	180	Mori and Mizuno, 2001a
ZmNAS2	AB061271	Root, leaf, seed endosperm	GCTATGTCACGGGCATCGCA GAACATTTGCTTGCGCAGGC	172	Mori and Mizuno, 2001b
ZmNAS3	AB042551	Root, leaf, seed endosperm	TCGCTCTTCCCGTACATCAA CCGCAGATGTCGTAGTTGTC	191	Mori and Mizuno, 2002

Soybean ferritin is the transgene used in this study.

### Comparison of zein proteins in maize seed endosperms positive or negative for the soybean ferritin transgene

This experiment was carried out to compare zein protein levels in maize seeds, positive or negative for the soybean ferritin transgene using the high performance liquid chromatography (HPLC). The materials used for this analysis were obtained from plants harvested in the third generation. Three transformation events (P344-2-4-1, P344-4-1-6, and P344-5-2-1) and three ears per event were used for this study. In brief, a 10 mg sample was drilled from each of the several kernels from the three ears from each of the three transformation events. DNA was extracted from each of the sample and tested with PCR to identify positive and negative maize seeds for the soybean ferritin transgene. The PCR reaction primers and conditions were as described in the previous sections. The remaining portion of the seed endosperm (positive or negative) was used to obtain another 10 mg sample for zein extraction and HPLC analysis.

#### Extraction of zein proteins and HPLC analysis

Maize zeins were extracted from each 10 mg sample with 400 μl of extraction buffer (70% Ethanol and 61 mM sodium acetate solution) in a 1.5 m centrifuge tube. The tubes were shaken for 1 h at room temperature before they were centrifuged for 10 min at 14,000 rpm. The supernatant was removed and transferred into a fresh tube. One hundred micro liters of supernatant were transferred into a vial (Alltech 8 × 30 mm shell vial clear glass, catalog 97029) and used for HPLC analysis using the Waters Alliance HPLC system. The solvents used were trifluoroacetic acid (TFA) (0.1%) in ultrapure water, TFA (0.1%) in acetonitrile (Paulis et al., 1991). Samples were separated with a c-18 column (Vydac 218TP54) and the UV absorbance was monitored at 210 nm. Levels of individual zeins were determined by integration of the resulting peaks.

### Comparison of total mineral concentrations in ferritin PCR positive and negative maize seeds

Previous studies have shown that activation of enzymes related to iron uptake also increases the uptake of other divalent metal cations (Welch, 1993; Delhaize, 1996; Vansuyt et al., 2000). In light of these previous findings we sought to carry out mineral quantification in maize seeds transformed with the soybean ferritin transgene. The minerals that were analyzed in this study included calcium, copper, magnesium, manganese, zinc and iron. Three transformation events (P344-2-4-1, P344-4-1-6, and P344-5-2-1) were analyzed and samples were obtained from three maize ears for each of the events. The analysis was done on ground whole maize seeds that tested positive or negative using PCR, for the soybean ferritin transgene. Samples of 1.5 g were prepared from 20–45 seeds (depending on kernel size) from a bulk of ears from the same transformation event. Six samples, three PCR positives and three PCR negatives were prepared for each of the transformation events. Samples were analyzed for mineral content using inductively coupled plasma atomic emission spectrometry (ICAP) at the Soil and Plant Analysis Laboratory, Iowa State University. A total of eighteen samples were analyzed in duplicates.

### Comparison of percent total nitrogen in maize seed samples positive or negative for the soybean ferritin DNA

Percent total nitrogen (as a measure of percentage total protein) was measured using the LECO CN combustion elemental analyzer (LECO Corporation, St. Joseph, MI) and using the same materials as those in the previous section on mineral analysis. As described above, samples negative or positive for the soybean ferritin transgene were compared in three transformation events (P344-2-4-1, P344-4-1-6, and P344-5-2-1).

### Statistical analysis

For zein and nitrogen data, analysis of variance (ANOVA) was carried out using JMP SAS-based statistical software (Version 8.0.1) in which the variance was partitioned among the effects listed in Tables 2 and 3 by least squares fitting to a linear model. All effects were considered fixed, limiting the inference space to observations in this experiment and allowing us to estimate the magnitudes and significance probabilities of all effects. The significance of the transgene PCR + or − effect indicates whether a significant difference was between transgenic and non-transgenic plants and is therefore the effect of greatest interest and wherever significant differences were obtained, means were compared by the student's *t*-test. For the qPCR analyses, the delta cycle threshold values were used and these were calculated as discussed in Pfaffl (2001).

**Table 2:** **Transcript ratios of transgene PCR positive plants relative to PCR negative plants averaged across four transformation events **(P344-2-4-1, P344-4-1-6, P344-5-2-1, and P344-6-6-1) in three tissues (roots, leaves, and seed endosperm) at two developmental stages (1 MAP and 18 DAP)****.

**Gene**	**Tissue**				
	**Root**		**Leaf**		**Endosperm**^**1**^
	**1 MAP**	**18 DAP**	**1MAP**	**18 DAP**	**18 DAP**
Transgene: Soybean ferritin^2^	n.a.	n.a.	n.a.	n.a.	32.21^***^
*ZmNAS1*	0.94	1.02	0.99	1.08	4.76^**^
*ZmNAS2*	0.93	1.02	0.98	0.90	5.88^**^
*ZmNAS3*	1.08	0.95	0.76	0.39	4.23^**^
Maize ferritin	1.10	1.82	1.10	1.04	4.13^*^
Yellow stripe 1	0.92	1.01	n.a.^3^	n.a.	n.a.
*ZmNAAT1*	4.35^**^	5.00^**^	n.a.	n.a.	n.a.
Maize ferredoxin	n.a.	n.a.	1.49	1.49	4.50^*^
4Fe-4S ferredoxin	n.a.	n.a.	1.08	6.67^*^	5.29^***^
27kDa γ-zein	n.a.	n.a.	n.a.	n.a.	1.36
16kDa γ-zein	n.a.	n.a.	n.a.	n.a.	1.05
15kDa β-zein	n.a.	n.a.	n.a.	n.a.	1.35
19kDa α-zein	n.a.	n.a.	n.a.	n.a.	8.34^**^
18kDa δ-zein	n.a.	n.a.	n.a.	n.a.	1.10
22kDa α-zein/Floury2	n.a.	n.a.	n.a.	n.a.	8.35^**^

1 Endosperm was examined only at 18 DAP because it was not formed yet at 1 MAP.

2 Since the transgene is not present in the PCR negative samples, these ratios are represent of the PCR positive samples to the background level.

3 n.a.: measurement not taken because genes are not expressed in tissue.

*, **, and *** indicate significance probabilities at P < 0.05, P < 0.001, and P < 0.0001, respectively.

**Table 3:** **ANOVA Table showing the sums of squares for the mean HPLC peak areas of maize zein proteins in maize seed samples positive or negative for the soybean ferritin transgene**.

**Model effects**							
		**Event**^**1**^	**Transgene PCR + or −**^**2**^	**Event × Transgene PCR + or −**	**Error**	**Total**
	**DF**^**3**^	**2**	**1**	**2**	**12**	**17**
HPLC Peak^4^	1	2.39E+12	9.87E+12^***^	1.16E+11	5.70E+12	1.81E+13
	2	4.88E+12	2.15E+12	2.74E+12	1.27E+13	2.24E+13
	3	7.91E+12	7.47E+12^*^	1.00E+12	1.23E+13	2.87E+13
	4	1.62E+11	3.47E+12^***^	2.93E+11	1.06E+12	4.90E+12
	5	1.79E+12	1.32E+13^**^	1.17E+12	1.14E+13	2.75E+13
	6	1.59E+11	5.27E+12^***^	4.53E+11	2.10E+12	7.98E+12
	7	9.40E+11	2.27E+13^**^	1.39E+12	8.82E+12	3.39E+13
	8	1.30E+13	1.885E+14^***^	1.61E+13	1.12E+14	3.29E+14
	9	1.45E+12	2.7973E+13^***^	9.25E+11	5.19E+13	1.08E+14
	10	1.84E+12	4.7691E+13^**^	6.14E+12	5.19E+13	1.08E+14
	11	3.96E+11	9.98E+12	4.25E+11	8.98E+11	1.17E+13
	12	5.11E+11	1.297E+11^**^	2.38E+11	1.13E+12	2.01E+12
	13	1.28E+12	3.3112E+12^***^	1.26E+12	2.02E+13	2.60E+13
	14	1.00E+12	1.39E+13	2.53E+12	1.49E+13	3.24E+13
	15	4.34E+09	3.3112E+11^*^	1.58E+11	9.45E+11	1.44E+12

Significant differences between PCR positive and negative seed samples are indicated with one, two, or three stars before each value to represent significant differences at 5, 0.1, and 0.01% significance levels, respectively.

1 Three events were used in this study: P344-2-4-1, P344-4-1-6 and P344-5-2-1.

2 Samples were screened with PCR to identify transgene-encoded ferritin positive and negative seeds.

3 Degrees of freedom. Read across row.

4 HPLC peak assignments are shown in Figure 7.

*, **, and *** indicate significance probabilities at P < 0.05, P < 0.001, and P < 0.0001, respectively.

## Results

### Expression of soybean ferritin tansgene in maize seed endosperm

**Figure 2:**
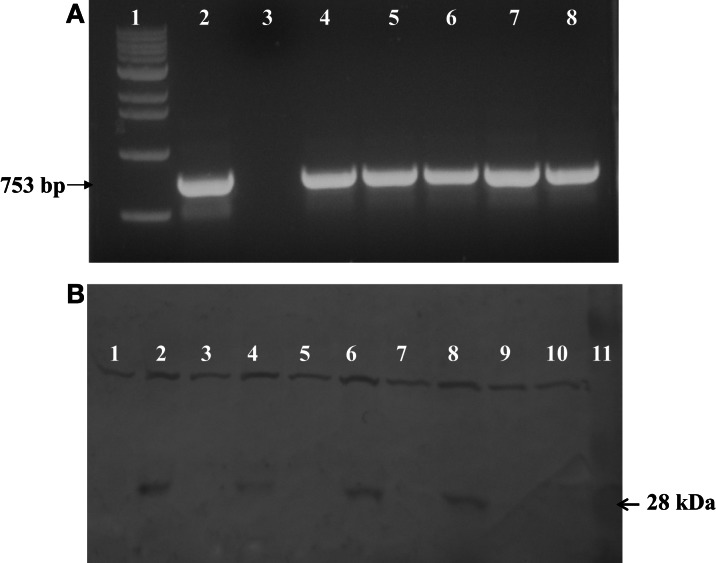
**(A)** One percent agarose gel stained with ethidum bromide showing PCR analysis results of soybean ferritin DNA detection in transformed maize lines and the non-transformed line B73 used as a negative control. Lanes 1 and 2 refer to 1 kb molecular DNA size marker and soybean ferritin plasmid (pMNK01) positive control, respectively. Lane 3 represents the negative control while lanes 4 through 8 represent the transformed maize lines (P344-2-4-1, P344-4-1-6, P344-5-2-1, P344-6-6-1, and P344-7-4-1, respectively). The black arrow shows the specific 753 bp fragment of soybean ferritin coding sequence. **(B)** Western blot showing the 28 kDa soybean ferritin protein band in maize seed endosperms. Lanes 1 through 8 represents transformed maize seeds that were PCR, negative (lanes 1, 3, 5, and 7) or PCR positive (lanes 2, 4, 6, and 8) for the soybean ferritin transgene. Each positive/negative pair of lanes are from a different transformation event (P344-2-4-1, P344-4-1-6, P344-5-2-1, and P344-6-6-1). Lanes 1 and 3 are from event P344-2-4-1; lanes 3 and 4 are from event, P344-4-1-6; lanes 5 and 6 are from event P344-5-2-1 and lanes 7 and 8 are from event P344-6-6-1. Lanes 9 and 10 non-transgenic A188 and B73 seeds that were used as negative controls, respectively, while lane 11 contains the protein molecular size marker.

PCR was used to test for the presence of soybean ferritin transgene DNA in maize leaves and seed endosperm. A band of the expected size was detected in some plants in the first through the fourth generation plants. This indicated successful integration and sexual transmission of the soybean ferritin transgene into the maize genome. The PCR product was not detected in non-transgenic B73, the negative control (Figure 2A). Of the 272 seeds tested from 6 transformation events of F_1_ plants, 141 were positive for the soybean ferritin transgene following PCR screening (Data not shown).

After DNA detection, we analyzed the ferritin protein expression using sodium dodecyl sulfate polyacrylamide gel electrophoresis (SDS-PAGE) and western blot analyses. As anticipated, the 28 kDa soybean ferritin protein was detected in PCR positive samples but not in the PCR negative samples implying that the transgene was effectively expressed in the maize seed endosperm. In addition to the targeted protein, the antibody used also cross-reacted with another protein of a higher molecular weight in both PCR positive and negative samples for the soybean transgene. No immune reactive 28 kDa band was detected in untransformed A188 and B73 samples (Figure 2B).

### Expression of endogenous genes in leaves, roots, and seed endosperm of maize transformed with the soybean ferritin transgene

In this experiment, differential expression of endogenous genes in roots, leaves, and seed endosperms was examined in maize lines transformed with the soybean ferritin transgene. Generally, the soybean ferritin transgene only affected expression of a few genes of those tested in maize leaves and roots measured during the vegetative and reproductive stages. The vegetative stage represents period before transgene expression (1 MAP) while reproductive stage represents period after transgene expression (18 DAP).

**Figure 3:**
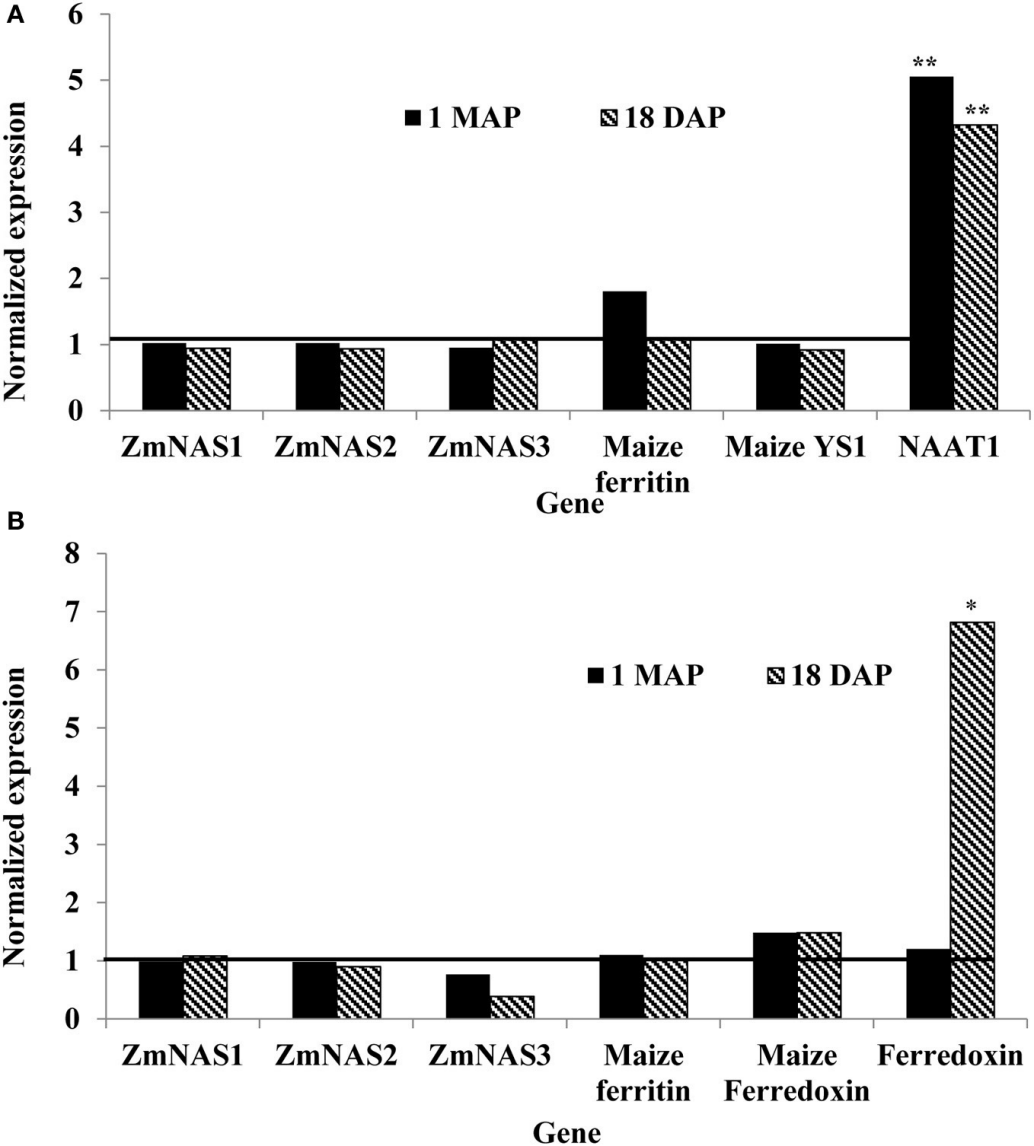
**Relative mRNA changes in maize plant roots (A) and leaves (B) at 1 month after planting (MAP) and 18 days after pollination (DAP).** Maize plants were selected based on presence and absence of the soybean ferritin DNA using PCR. Data collected at 1 MAP and 18 DAP represent time before and after soybean ferritin transgene expression, respectively. For each gene, the delta cycle threshold (ct) values were normalized using 18S RNA. mRNA transcript levels in both PCR negative and positive samples were compared after normalization with respect to the ferritin PCR negative samples. The horizontal line on Figure represents values for PCR negative samples, normalized to one relative to the PCR negatives. Significant differences at *P* < 0.05 and *P* < 0.001) between transgene PCR positive and negative samples are indicated with one star and two stars, respectively, on top of the bar.

In maize roots, the only significant differences (*P* = 0.0303 and *P* = 0.0097; vegetative and reproductive stages, respectively) in transcript levels were observed for the *NAAT1* gene between the PCR positive and negative plants (Figure 3A). PCR positive plants had 4.35 and 5 times more *NAAT1* mRNA before and after soybean ferritin transgene expression, respectively, relative to the PCR negative plants (Table 2). The transcript accumulation of other genes (*ZmNAS1, ZmNAS2, ZmNAS3, maizeYS1*, and maize ferritin) did not differ significantly (*P* > 0.05) between soybean ferritin PCR negative and positive maize root samples (Figure 3A; Table 2). Comparing expression levels of all the genes tested relative to 18S RNA, we observed that *ZmNAS2, NAAT1, ZmNAS1*, and *Maize YS1* were the most highly expressed while *ZmNAS3* and maize ferritin were the least expressed before transgene expression (Data not shown). After transgene expression, the transcript level of maize ferritin increase along the others but *ZmNAS3* did not change. In either case, however, it was only *NAAT1* that was significantly higher in transgene PCR positive samples compared to the control.

Gene expression results from leaf samples were almost identical to those observed from the root samples. Prior to transgene expression, there were no significant differences in mRNA levels of all the tested genes between transgene PCR positive and negative samples (Figure 3B). However, after transgene expression, the amount of ferredoxin binding protein mRNA levels significantly (*P* < 0.05) increased 6.67 fold in transgene PCR positive samples compared to the PCR negative samples (Figure 3B; Table 2). Comparing changes in the expression levels of all genes relative to 18S RNA showed that *ZmNAS1* had the highest mRNA levels compared to other genes during the vegetative stage, while maize ferritin and maize ferredoxin and ferredoxin binding protein were highly expressed compared to others during the reproductive stage.

**Figure 4:**
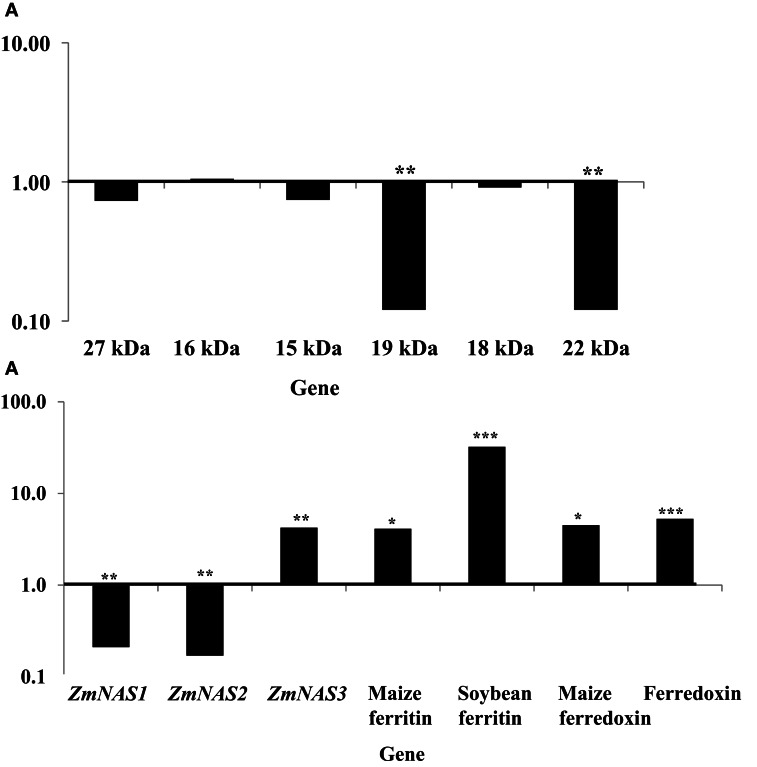
**mRNA transcript changes in maize seed endosperm samples harvested at 18 days after pollination (DAP).** Maize seed endosperms were selected based on presence and absence of the soybean ferritin DNA. The 18 DAP represents the time after the soybean ferritin transgene expression. Panels **(A)** and **(B)** represent qPCR analysis results for the maize seed storage protein genes (zeins) and iron homeostasis genes, respectively. For each gene, the delta cycle threshold (delta ct) values were normalized using 18S RNA, with respect to the ferritin PCR negative samples. The horizontal lines on the panels represent normalized values for the PCR negative samples. Significant differences (*P* < 0.0001, *P* < 0.001, and *P* < 0.05) are indicated with three, two, and one star(s), respectively, on top of each bar for which the soybean ferritin PCR positive and negative samples differ.

More dramatic expression differences were observed for genes endogenous to maize seed endosperm (Figures 4A,B). The presence of soybean ferritin transgene in the seed endosperm significantly (*P* < 0.001) reduced mRNA accumulation of the 19 and 22 kDa zeins but did not affect (*P* > 0.05) mRNA levels of the 27, 16, 15, and 18 kDa zeins (Figure 4A). The 19 and 22 kDa zeins mRNA was reduced 8.34 and 8.35-fold, respectively, in PCR positive samples compared to the negative ones (Table 2). Similar to the zein transcripts, the mRNA abundance of the seven tested iron homeostasis genes differed between seed samples positive and negative for the soybean ferritin transgene in the maize seed endosperm. The samples positive for the transgene had significantly (*P* < 0.0001) more transcripts of the soybean ferritin, ferredoxin ferrodoxin, maize ferredoxin, maize ferritin, and *ZmNAS3* genes than the negative samples (Figure 4B). Ferredoxin, maize ferritin, maize ferredoxin and *ZmNAS3* showed atleast four times more expression in the soybean ferritin PCR positive seed endosperms than in the negative ones (Table 2). On the contrary, the presence of the transgene significantly (*P* < 0.001) reduced the accumulation of *ZmNAS1* and 2 mRNA in PCR positive seed endosperms compared to their negative counterparts (Figure 4B).

When we analyzed mRNA accumulation levels of all zein genes relative to 18S RNA, results showed that the 16 kDa was the most highly expressed compared to all other genes. However, the analysis of iron homeostasis genes' mRNA accumulation in soybean ferritin PCR positive and negative samples relative to 18S RNA showed that *ZmNAS2* had significantly (*P* < 0.0001) higher expression levels compared to all other genes, although significant differences were also observed among individual PCR positive and negative samples (Data not shown).

### Zein protein profiles in maize, positive and negative for the soybean ferritin transgene

**Figure 5:**
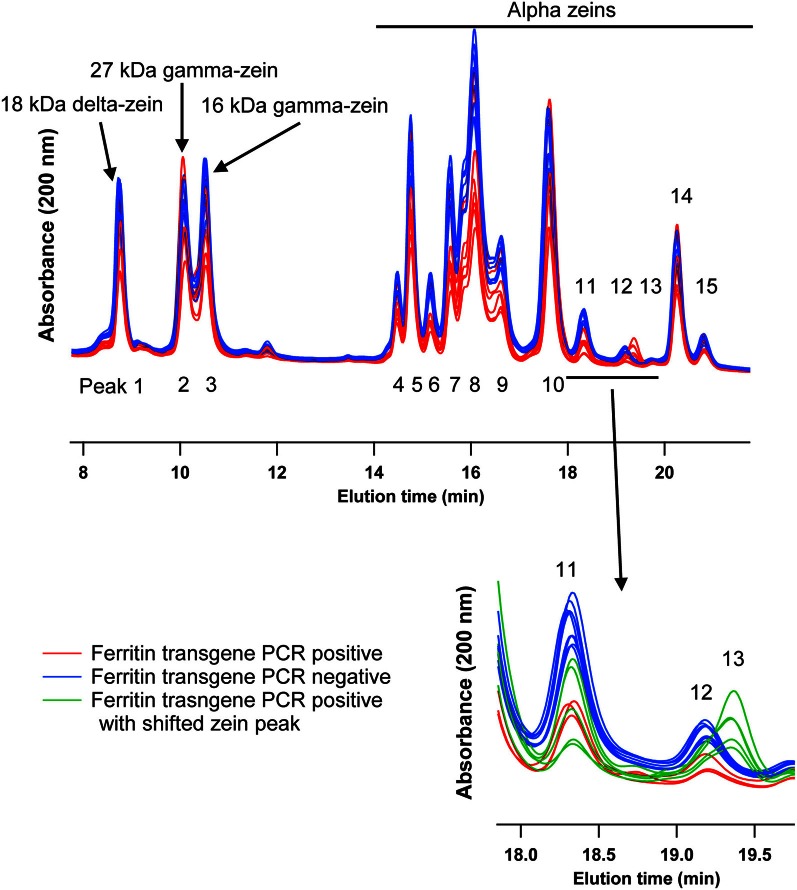
**HPLC chromatograms of maize zeins for maize seed endosperms transformed with the soybean ferritin transgene.** Maize seed endosperms were screened with PCR to identify soybean ferritin positive (Red or bottom) and negative (blue or top) seed samples. Samples were separated with a c-18 column. Peaks are labeled 1–15, depending on elution time. When known, the peaks (1, 2, and 3) are labeled with their respective sizes (kDa) and 4–15 refer to the unassigned alpha zeins. The inset emphasizes a peak that appeared in some transgene PCR positive samples, peak 13 in green traces.

**Figure 6:**
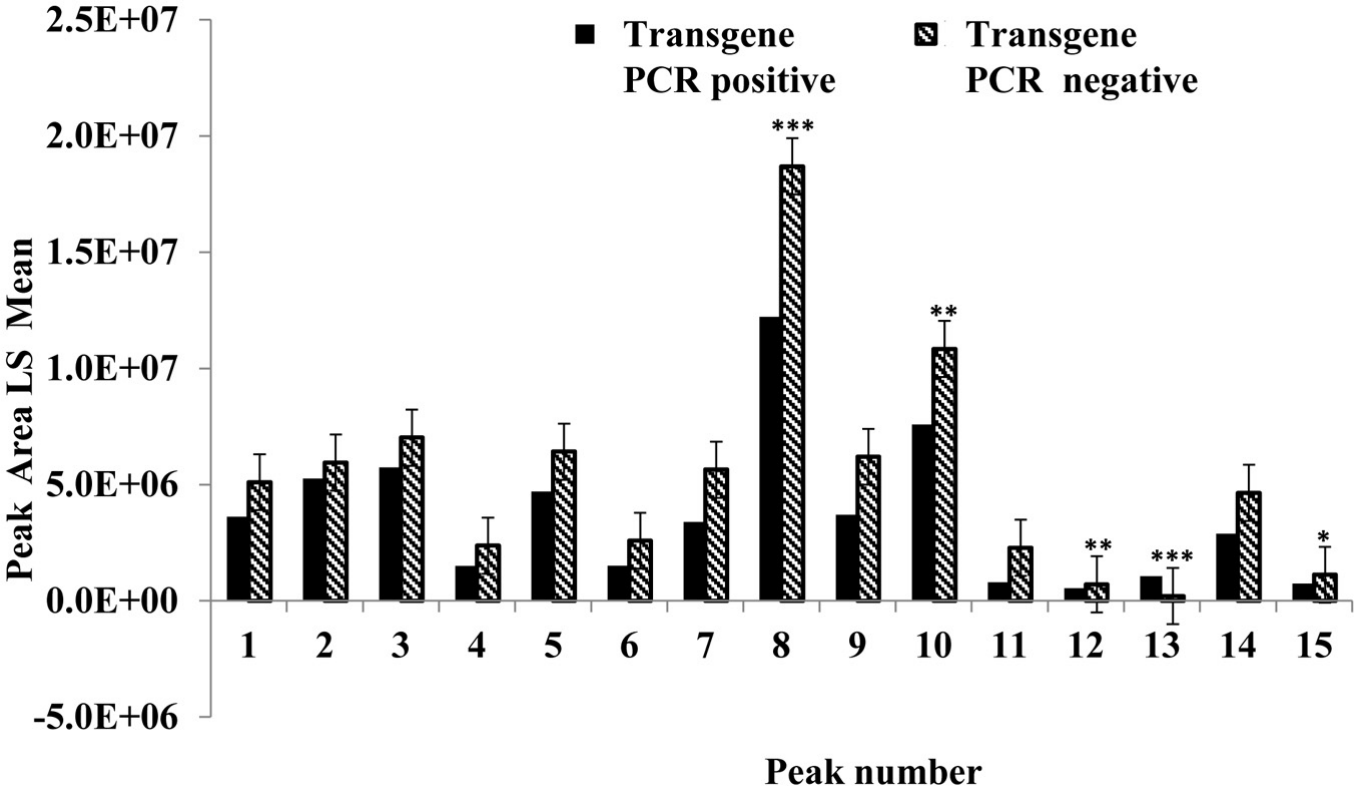
**Peak area least square means for the maize seed storage protein genes (zeins).** The horizontal axis shows the HPLC protein peaks, named 1–15 depending on the elution time (see Figure 5). Significant differences between transgene-encoded ferritin PCR positive and negative maize seed samples are indicated with one, two, and three stars on top of each bar to represent significant differences at 5, 1, and 0.1% significance levels, respectively.

**Figure 7:**
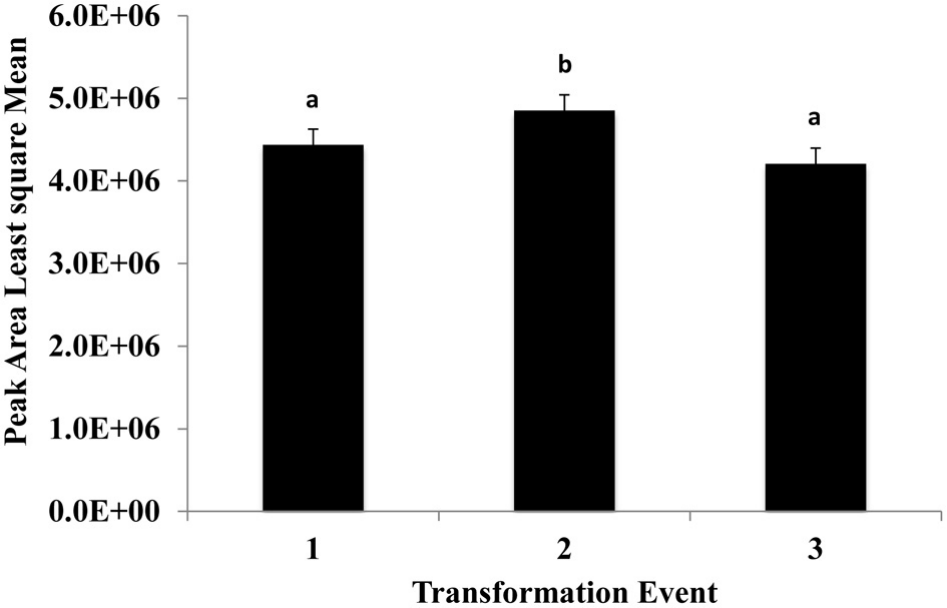
**The effect of three transformation events, 1, 2, and 3 (P344-2-4-1, P344-4-1-6, and P344-5-2-1, respectively) on mean peak areas of maize seed storage protein genes for maize seed endosperms transformed with the soybean ferritin transgene.** Significant differences (*P* < 0.05) are indicated with a different letter on top of each error bar.

This experiment was carried out to determine if presence of the soybean ferritin transgene affected the overall profile of zein proteins in the transformed maize. The chromatograms showed some qualitative and quantitative differences between soybean ferritin PCR positive and negative samples (Figure 5 and Table 3). Zein profiles for each group are given numbers (1–15) depending on the elution time (peaks). For already described zeins i.e., 18δ, 27γ, and 16γ zeins, individual protein peaks were identified and their molecular sizes (kDa) indicated. Most of the peaks within the PCR negative and positive samples had similar elution patterns, indicating same protein profile in PCR negative and positive samples (Figure 5). However, in some of the PCR positive samples, the elution time for peak 12 was shifted resulting in position 13 (see expanded portion of the chromatogram, Figure 5). The occurrence of samples whether positive or negative for the transgene influenced the magnitude of the mean peak area. The peak areas in PCR negative samples were significantly (*P* < 0.0001) higher than their PCR positive counterparts. This was true mostly for the alpha zeins (area peaks 4–15) while the relative proportions of the delta (area peak 1) and the gamma (peaks 2 and 3) zeins were similar within the PCR positive and negative seed endosperm samples (Figure 6). Alpha peaks 8, 10, 12, 13, and 15 had significantly higher peak areas in PCR negative samples compared to the positive samples (Figure 6). The mean differences between peak areas for events one and two were significantly (*P* < 0.05) different among PCR positive and negative samples with the PCR negative samples having higher peak areas in all the transformation events (Figure 7).

### Effects of the soybean ferritin transgene on mineral compostion in the maize seed endosperm

**Figure 8:**
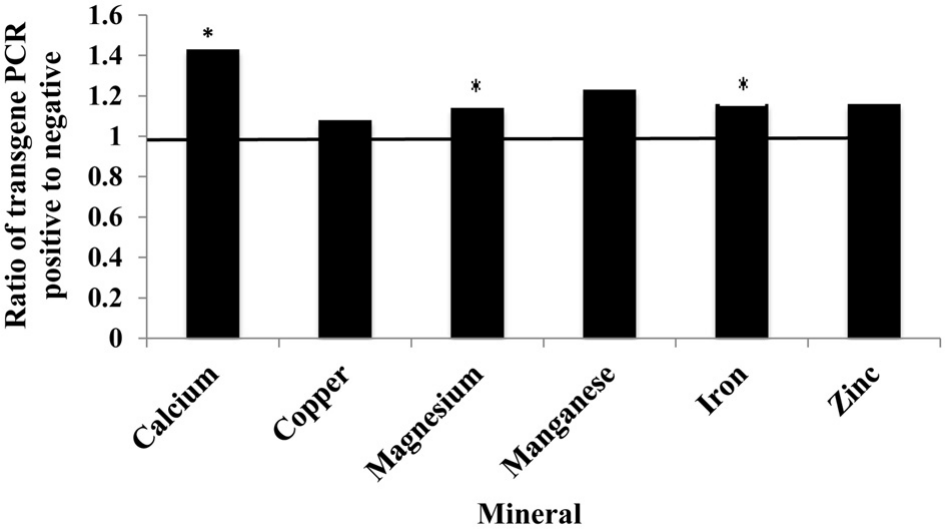
**Fold change in mean mineral concentration in maize seed samples transformed with the soybean ferritin transgene.** The samples were screened with PCR to categorize them into those positive and negative for the soybean ferritin transgene. Three transformation events (P344-2-4-1, P344-4-1-6, and P344-5-2-1) were used for this test. The event effect was not significant (data not shown), therefore, the results presented are from combined data. The horizontal line represents normalized values for the transgene PCR negative samples. Significant differences (*P* < 0.05) are indicated with a star on top of each error bar for which the PCR positive and negative samples differ.

The presence of the soybean ferritin transgene significantly (*P* < 0.05) affected the concentration of calcium, magnesium, and iron but not copper, manganese, and zinc. The mineral concentrations of calcium, magnesium and iron were higher in samples positive for the soybean ferritin transgene compared to the negative samples (Figure 8). Although the iron concentration in samples positive for the transgene differed significantly from that in the negative samples, they were only 0.2 times higher. While the mean mineral concentrations of copper, manganese and zinc were higher in the soybean ferritin PCR positive samples compared to the negative samples, their mean differences were not significant at *P* = 0.05.

### Percent total protein decreased in maize seed endosperms transformed with the soybean ferritin transgene

The mean percentage total protein differences between PCR positive and negative maize seed endosperm samples were highly significant (*P* < 0.0001) (Table 4). Percentage total protein was higher in transgene PCR negative samples than PCR positive samples. On the other hand, transformation events (P344-2-4-1, P344-4-1-6, and P344-5-2-1) had significant differences in total nitrogen measurements between samples positive and negative for the soybean ferritin transgene (Table 4). Transformation events 1 (P344-2-4-1) and 3 (P344-5-2-1) had significantly higher total nitrogen (1.678 ± 1.354% and 1.666 ± 1.354%, respectively) compared to transformation event 2 (P344-4-1-6) (1.592 ± 1.354%). In this experiment, the presence of the soybean ferritin transgene was associated with decreased percentage total protein in maize seed endosperms.

**Table 4:** **ANOVA for the least square means for percentage total nitrogen in transformed maize seeds, positive or negative for the soybean ferritin transgene**.

**Least square area means (total nitrogen)**				
**Effect**	**DF**^**1**^	**Sums of squares**	***F*-ratio**	**Prob > *F***
Replicate	1	0.0022	0.3471	0.5613
^2^Transgene PCR + or −	1	0.4984	73.0392	<0.0001^***^
^3^Event	2	0.0525	3.8465	0.0356^*^
Transgene PCR + or − × replicate	1	0.0007	0.1095	0.7436
Transgene PCR + or − × Event	2	0.0194	1.4206	0.2612
Event × replicate	2	0.0064	0.4696	0.6309
Transgene PCR + or − × Event × replicate	2	0.0028	0.2080	0.8137

1 Degrees of freedom.

2 The transformed maize seed samples were screened with PCR to identify those positive and negative for the soybean ferritin transgene. Comparisons are made based on significant differences in percentage total nitrogen between transgene PCR positive and negative samples.

3 Three transformation events were examined in duplicate for this test (P344-2-4-1, P344-4-1-6, and P344-5-2-1).

* and *** indicate significance probabilities at P < 0.05 and P < 0.0001, respectively.

## Discussion

Increasing the nutritive value of plants for human benefit has been an area of interest to many research groups. Maize is an excellent target crop because it serves as a major dietary source to many nations, especially those in the developing world, who cannot afford the cost of food supplements. The main objective of this study was to determine the effect of the soybean ferritin transgene on endogenous gene transcripts and protein levels in maize roots, leaves, and seed endosperms. Maize plants expressing the soybean ferritin gene directed to the seed endosperm by the super gamma zein promoter were produced. Soybean ferritin DNA was successfully detected in the leaves and seed endosperm samples, with a corresponding increase in the transgene protein expression in only the ferritin PCR positive seed samples, indicating a successful transformation and integration process. One of the strategies for alleviating mineral or iron deficiency health problems is to produce transgenic plants with increased ferritin levels as reported in findings by different research groups (Goto et al., 1999; Brinch-Pedersen et al., 2000; Drakakaki et al., 2000, 2005; Lucca et al., 2001, 2002; Vasconcelos et al., 2003; Qu et al., 2005; Aluru et al., 2011).

Transgene introduction in plants can potentially lead to unintended modifications in transcription and translation of native genes in the host plants. It is therefore necessary to assess the likelihood of these occurrences by measuring transcript changes of native genes especially those in tissues where the transgene is inserted or is likely to cause significant changes. Transgene insertion into the host genome is a random process which can result in the transgene landing in the coding sequence of a native gene. When this happens, it can cause changes in the stability or levels of mRNA accumulation of the native gene. Therefore, one of the ways in which we can determine whether or not the transgene causes some accidental effects is to look at mRNA levels of native genes that control the routine activities or that are directly involved in the regulation of specific metabolic processes where the transgene is to be acting. We made an effort in this study to assess the effects of soybean ferritin transgene on the transcript accumulation of native genes in maize roots, leaves and seed endosperm and at two developmental stages. Our results showed that the presence of the soybean ferritin transgene had no significant effects (P > 0.05) on most of the genes in the maize roots and leaves, before (1 MAP) and after (18 DAP) transgene expression in the target tissue. Measurement of mRNA transcripts for *ZmNAAT1* gene, however, showed significantly (*P* < 0.05) higher mRNA amounts for the positive PCR root samples than the negative ones at the two data collection points. *ZmNAAT1* is an enzyme that is required in the homeostasis of mugineic acid (MA), an important metal chelator that is involved in the transportation of metal ions, including iron ions across the plasma membrane in both grass and non-grass plants (Higuchi et al., 1996). In rice, Cheng et al. (2007) reported that a mutation in the rice *NAAT1* gene led to substantial accumulation of nicotianamine in roots and shoots but the mutant plants could not effectively absorb Fe (III) because of the failure to produce deoxymugineic acid. Nicotianamine is a chelator that occurs in plants and it is involved in internal iron transport (Stephan et al., 1994, 1996). In our experiment, in maize roots, presence of the soybean ferritin transgene was associated with increased levels of *ZmNAAT1* mRNA. This increase would make sense if the plant was experiencing iron deficiency because iron was sequestered in the seed in the transgene-encoded ferritin. It has been reported that overexpression of soybean ferritin in transgenic tobacco caused plants to respond as if they were iron deficient due to illegitimate sequestration of iron (Van Wuytswinkel et al., 1999; Vansuyt et al., 2003). This explanation is not completely satisfying, however, because the *ZmNAAT1* transcript is up-regulated in transgenic plants prior to seed set as well as after seed set. Our results are in contrast to those of Aluru et al. (2011) who found that in roots of maize plants transformed with the same transgene used in this study, NAAT mRNA was reduced two to four fold. Accumulation of this message may therefore be influenced by the environment or the genetic background of the plants used in the study. Earlier studies also indicated that the presence of iron increased the amount of ferritin mRNA in bean leaves and soybean cell cultures by more than 30 fold (Proudhon et al., 1989). The fact that transcript levels of other genes in maize roots were not different in presence or absence of the soybean ferritin transgene illustrates that there was no detectable interaction between these genes and the soybean ferritin transgene in these tissues.

Gene expression results from leaf samples were almost identical to those observed with the root samples, with only the ferredoxin or Iron–sulphur (4Fe-4S) proteins showing increased transcript levels in samples where the soybean ferritin transgene was detected. 4Fe-4S proteins contain iron and sulphur bound to the polypeptide chain by 4 cysteinyl sulfur linkages to the iron atoms (Sweeney and Rabinowitz, 1980). These proteins are well-known for their involvement in electron transport and gene expression regulation (Lill and Muehlenhoff, 2006). It is likely that the increased transcription of ferritin, an iron storage protein, led to increased transcript levels of this gene. Because the amount of iron present in a given tissue regulates the transcription of the ferritin gene (Savino et al., 1997; De Domenico et al., 2008), it is possible for the iron to similarly have the same effects on genes that act in the same pathway as the ferritin gene and this could explain the relative increase in the transcripts of the genes in samples where the ferritin transgene was detected.

Different endogenous maize genes showed differential gene expression patterns in maize seed endosperm samples with or without the soybean ferritin transgene. Two of the alpha zein protein transcripts (19 and 22 kDa α-zeins) were significantly lower in transgenic plants than in non-transgenic plants. We found more than 8-fold decrease in mRNA accumulation in the 19 and 22 kDa in the PCR positive samples. Conversely, no significant mRNA changes were observed in the gamma and delta zeins as well as other alpha zein proteins tested. These results were consistent with those obtained in the samples harvested and analyzed in earlier previous year (data not shown). These observations are also supported by the zein protein analysis results that showed increased accumulation of these same proteins in soybean ferritin PCR negative compared to PCR positive samples. In addition, the percent total nitrogen levels were also significantly higher in ferritin PCR negative samples compared to the PCR positive samples. The use of the super gamma zein promoter is a possible explanation for the observed differences. This promoter shares regulatory sequences with endogenous genes and could compete for transcription factors, which could result in reduction in endogenous gene transcription levels. It is somewhat surprising that differences were not observed in the zein most closely related to the transgene, the 27 kDa gamma zein, but were observed in other zeins. Peak 13 was present only in PCR positive samples and these samples lacked Peak 12. We don't know the identity of this peak, but it's co-occurrence with the transgene is intriguing. Several of the alpha peaks had significantly higher peak areas in PCR negative compared to the positive samples. The amount of zein protein depended on both transformation event and presence or absence of the soybean ferritin transgene in the maize. In fact, measurement of the percent total protein in maize seeds without the soybean ferritin transgene showed that they had significantly higher percent total protein than their corresponding positive ones. The high values for zein transcript and protein levels in soybean ferritin negative samples are consistent with percentage total protein content results.

On the other hand, transcripts of iron homeostasis genes in maize seed endosperm accumulated to higher levels in ferritin PCR positive samples compared to their negative counterparts. The regulation of iron homeostasis genes depends on the iron status in the tissue of interest (Savino et al., 1997; De Domenico et al., 2008). It has been reported that overexpression of soybean ferritin in transgenic tobacco caused plants to respond as if they were iron deficient due to an illegitimate sequestration of iron (Van Wuytswinkel et al., 1999; Vansuyt et al., 2003). Thus, changes seed ferritin levels will most likely correlate with up or down-regulation of iron homeostasis genes. In this study, the mRNA transcripts of the soybean ferritin gene accumulated to high levels in maize endosperm samples. In plants, ferritin production is regulated at both transcriptional and post transcriptional levels (Lescure et al., 1991; Kimata and Theil, 1994; Savino et al., 1997). This is dependent on presence of iron that results in the relative induction of ferritin mRNA and protein levels. Protein analysis revealed soybean ferritin protein accumulated to detectable levels only in PCR positive samples. Kimata and Theil (1994) reported stable ferritin mRNA levels in soybean plants during development, with a corresponding decrease in ferritin protein content. Measurement of iron levels in maize seed endosperm samples indicated a direct correlation between presence of ferritin transgene and iron concentrations. The soybean ferritin PCR positive samples in general contained higher mineral concentrations with significant differences observed for calcium, magnesium and iron. The amount of iron that accumulated in PCR positive samples was 0.2 fold higher than in the PCR negative samples. Previous studies (Goto et al., 1999; Vasconcelos et al., 2003; Drakakaki et al., 2005) reported even higher correlations between soybean ferritin expression levels and total iron content. On the contrary, Drakakaki et al. (2000), observed no significant increases in seed iron content in rice and wheat samples transformed with a ferritin gene and whose expression was controlled by the ubiquitin promoter. Iron concentration levels have been reported to differ from one generation to another (Qu et al., 2005) and this could possibly have happened in this case. The fact that different lines will likely have differences in their expression patterns could be another reason why we might expect differences in iron levels. However, in our findings, the different transformation events did not have significant effects on the amount of iron obtained. Changes in environmental factors have also been reported to influence iron uptake and storage (Vansuyt et al., 2000), a factor we cannot rule out in explaining this observation.

Significant increases in the concentrations of other metals (magnesium and calcium) in ferritin PCR positive seed samples could be due to increased iron accumulation resulting from the activation of the ferritin transgene. Vasconcelos et al. (2003) reported a similar finding in rice seeds with enhanced expression levels of the ferritin transgene. Previous research groups (Vansuyt et al., 2000; Welch, 1993) showed that it was possible to increase the uptake of other divalent metal cations with the activation of enzymes involved in iron uptake and use. Since other main factors and their interactions did not seem to have significant effects on metal accumulation, it seems very likely that the observed differences in metal concentrations were due to changes in iron status in maize seed samples. Qu et al. (2005), however, reported no significant changes in concentrations of calcium, copper, cadmium, magnesium, manganese, and zinc, a result that is contrary to our observations.

## Conclusion

This work describes efforts to assess the effect of soybean ferritin transgene on transcript and protein levels of maize endogenous genes. Differential expression patterns were observed between maize samples with or without the soybean ferritin transgene. The iron homeostasis genes selected for this study were up-regulated in soybean ferritin PCR positive samples compared to negatives ones. Only two of the zein protein genes were down-regulated in soybean ferritin PCR positive samples while the rest of the zeins remained unchanged. The transformation event did not affect transcript or protein levels of the different genes, but some genes were affected by the presence or absence of the transgene. By knowing transgene effects that can lead to changes in endogenous gene activities and levels, researchers will be able to find viable explanations for the unexpected results and be able to target specific stages controlled by specific genes in iron homeostasis.

### Conflict of interest statement

The authors declare that the research was conducted in the absence of any commercial or financial relationships that could be construed as a potential conflict of interest.
